# Exploring the Impact of Different *Saccharomyces cerevisiae* Strains on the Flavor Profile of Greengage Alcoholic Beverage Using GC-E-Nose, HS-GC-IMS, and HS-SPME-GC-MS

**DOI:** 10.3390/foods13243984

**Published:** 2024-12-10

**Authors:** Zhenbao Shi, Wenzhe Li, Ziqiang Chen, Zhengcong Peng, Mingtao Ma, Jinglong Zhang, Dianhui Wu, Guangfa Xie, Jian Lu

**Affiliations:** 1 Key Laboratory of Industrial Biotechnology of Ministry of Education, School of Biotechnology, Jiangnan University, Wuxi 214122, China; 6230210024@stu.jiangnan.edu.cn (Z.S.); wenzheli895@gmail.com (W.L.); 6220209065@stu.jiangnan.edu.cn (Z.C.); sd3961471@126.com (Z.P.); 6220201085@stu.jiangnan.edu.cn (M.M.); 6230210093@stu.jiangnan.edu.cn (J.Z.); 2 Key Laboratory of Pollution Exposure and Health Intervention of Zhejiang Province, College of Biology and Environmental Engineering, Zhejiang Shuren University, Hangzhou 310015, China; 3 National Engineering Research Center of Cereal Fermentation and Food Biomanufacturing, Jiangnan University, Wuxi 214122, China; jlu@jiangnan.edu.cn; 4 Jiangsu Provincial Research Center for Bioactive Product Processing Technology, Jiangnan University, Wuxi 214122, China

**Keywords:** greengage alcoholic beverage, volatile organic compounds, *S. cerevisiae*, GC-E-Nose, HS-SPME-GC-MS, HS-GC-IMS

## Abstract

Greengage alcoholic beverage (GAB) is gaining popularity for its distinctive fruity aroma and nutritional benefits. The *Saccharomyces cerevisiae* strain applied for fermentation significantly influences the metabolic production of aroma compounds, making the selection of *S. cerevisiae* strains to be critical for enhancing the unique flavor profile of the GAB in commercial production. In this study, the effects of two common commercial *S. cerevisiae* strains (SY, EC-1118) and two laboratory-derived strains (ET008-c54, NM-8) on the flavor profile of the GAB were investigated. Volatile organic compounds (VOCs) of the GAB samples were analyzed using GC-E-Nose, HS-SPME-GC-MS, and HS-GC-IMS. Results showed significant differences in the flavor compound content of the GAB fermented by different strains. Nine VOCs were identified as potential markers by partial least squares–discriminant analysis (PLS-DA) combined with variable importance in projection (VIP), with ethyl esters playing a key role in distinguishing the aroma characteristics of different groups. Consequently, 13 aromatic compounds were selected and considered to be the typical VOCs of the GAB. Notably, β-ionone and ethyl octanoate emerged as representative aroma compounds, both showing the highest concentrations in the SY sample. These findings will provide valuable insights into the aroma differences of the GAB fermented by different strains and offer theoretical guidance for selecting suitable strains in the industrial production of the GAB.

## 1. Introduction

As a deep-processing product of greengage, the fermented greengage alcoholic beverage (GAB) has emerged as a novel drink that aligns with modern health concepts due to its unique aroma, nutritional value, and functional characteristics [1]. Flavor is the essence of the fermented alcoholic beverage and serves as a critical indicator influencing consumer acceptance. The factors affecting the flavor quality of the fermented alcoholic beverage primarily include raw materials, fermentation processes, brewing *S. cerevisiae* strains, and storage conditions [2]. Among these, the interaction between the brewing *S. cerevisiae* and the fermentation liquid is crucial for flavor development. The complex interactions between *S. cerevisiae* and various substances in the fermentation liquid contribute to the appearance, taste, and flavor characteristics of the fermented alcoholic beverage [3]. The flavor compounds that form the basic framework of the aroma of the fermented alcoholic beverage include flavor substances converted from bound precursors during the *S. cerevisiae* metabolism and those synthesized during fermentation [4]. These compounds encompass higher alcohols, esters, acids, ketones, aldehydes, and terpenes generated by *S. cerevisiae* metabolism [5]. The types and concentrations of these substances play a significant role in enhancing the quality of the fermented alcoholic beverage [3]. Therefore, selecting the brewing *S. cerevisiae* that can highlight the unique flavor of the GAB is critical to improve the industrial quality of the GAB.

The aroma quality assessment of fermented fruit alcoholic beverages often relies on traditional sensory evaluation methods, which are criticized for their strong subjectivity. Therefore, it is crucial to develop a rapid, stable, highly sensitive, and cost-effective analytical method for evaluating the flavor characteristics of the GAB. Gas chromatography electronic nose (GC-E-Nose) is an intelligent sensing technology that simulates human olfactory functions and offers advantages in rapid analysis [6]. However, it has certain limitations in the precise identification of volatile organic compounds (VOCs). Headspace solid-phase microextraction gas chromatography–mass spectrometry (HS-SPME-GC-MS) is currently a widely used technique for food flavor analysis, capable of accurately separating and identifying VOCs in complex samples, while also allowing for further analysis using statistical methods. Therefore, HS-SPME-GC-MS is also a common flavor analysis technique for fermented fruit alcoholic beverages. Zhang et al. [7] characterized the flavor components of navel orange wine fermented by different *S. cerevisiae* and mixed fermentation conditions using HS-SPME-GC-MS, identifying a total of 78 VOCs. Cai et al. [8] identified 182 VOCs from red date wine fermented using different treatment methods with HS-SPME-GC-MS and selected differential metabolites based on the results of partial least squares–discriminant analysis (PLS-DA). However, the limitations of GC-MS, such as suboptimal resolution and reduced peak capacity, hinder its ability to detect trace concentrations of potential volatile compounds with low odor thresholds. The emergence of gas chromatography–ion mobility spectrometry (GC-IMS) addresses this limitation, offering high sensitivity and ease of operation, as well as the ability to visualize differences and similarities between samples through system plugins [9]. This technology has been applied in various areas, including evaluating aroma changes during fruit ripening, classifying spices and dairy products, and monitoring tea processing [10,11,12,13]. Liu et al. [14] utilized GC-IMS and GC-MS to profile the aroma compounds in yellow-fleshed peach wine, revealing that GC-IMS has a comparative advantage over GC-MS in detecting trace levels of aldehydes and ketones. Chi et al. [10] characterized flavor compounds in raw milk from different regions of China using a combination of GC-IMS and GC-MS, where the less abundant compound Ethyl trans-2-butenoate was detected solely by GC-IMS and served as one of the markers for distinguishing raw milk from different origins. Nevertheless, the lack of a comprehensive database for HS-GC-IMS restricts its application in the precise quantification analysis of samples. Therefore, the combined application of multiple flavor analysis techniques can compensate for each one’s limitations, providing a more comprehensive and reliable insight into the aroma information of fermented products.

This study conducted a comprehensive and detailed analysis of the GAB fermented by different *S. cerevisiae* strains using GC-E-Nose, HS-SPME-GC-MS, HS-GC-IMS, and sensory evaluation. Given the high-acid environment of greengage fruit fermentation [15], the study employs two laboratory strains of acid-tolerant *S. cerevisiae* strains and two commercially available *S. cerevisiae* strains commonly used for high-acid fruit fermentation [16,17,18,19]. Multivariate statistical analysis is applied to investigate the flavor differences among the GABs fermented by different strains. Moreover, the combined analysis of odor activity values (OAV) is utilized to identify key aroma compounds in the GAB. The aim is to select *S. cerevisiae* strains that can highlight the unique flavor of the GAB, providing theoretical guidance for the selection of *S. cerevisiae* in the industrial production of the GAB.

## 2. Materials and Methods

### 2.1. Samples and Chemicals

*S. cerevisiae* strains SY and EC-1118 were purchased from Angel Yeast CO., Ltd. (Yichang, China) and Lallemand CO., Ltd. (Montreal, QC, Canada), respectively. *S. cerevisiae* strains ET008-c54 and NM-8 were screened for the fermentation of fruit wine with a high content of acid and preserved in our laboratory. Greengage fruits were supplied by Liuliu Orchard Group Co., Ltd. (Wuhu, Anhui, China). Pectinase was purchased from Aladdin Biochemical Technology Co., Ltd. (Shanghai, China). Yeast extract, peptone, and glucose were purchased from Shanghai Aladdin Biochemical Technology Co., Ltd. (Shanghai, China). HCl, NaOH, CuSO_4_, NaKC_4_H_4_O_6_, and K_4_[Fe(CN)_6_] were of analytical grade and were obtained from Sinopharm Chemical Reagent Co., Ltd. (Shanghai, China).

### 2.2. Fermentation of Greengage Alcoholic Beverage

The fermentation of the GAB was conducted according to the method described by Tian et al. [20]. The fermentation conditions for the GAB were as follows: 200 g of greengage was crushed, with a solid-to-liquid ratio of 1:2.0 (g:mL); 200 g·L^−1^ of sugar was added to achieve an initial total sugar concentration of approximately 220 g·L^−1^, with pectinase at 36 U·g^−1^ and potassium bisulfite at 60 mg·L^−1^. A yeast in inoculation concentration of 1 × 10^7^ CFU·mL^−1^ was added, with a fermentation temperature of 20 °C.

### 2.3. Measurement of Physicochemical Properties

The total sugar and total acid contents were measured according to the standards in GB/T 15038 [21], while the alcohol content was determined following GB 5009.225 [22]. The color intensity and tonality of the GAB were assessed using a T6 UV-visible spectrophotometer (Beijing Purkinje GENERAL Instrument Co., Ltd., Beijing, China), according to the method outlined by Qiu et al. [23].

### 2.4. Sensory Analysis

The sensory evaluation panel consisted of 10 trained members (5 males and 5 females, aged 20 to 50 years). Participants assessed the GAB samples based on aroma, typicality, flavor persistence, overall acceptability, and color. Each attribute was scored on a scale from 0 to 9, where 0 indicated extremely weak and 9 indicated extremely strong. Pure water was provided for rinsing between the evaluations of different samples to avoid carry-over effects.

### 2.5. GC-E-Nose Analysis

GC-E-Nose (Alpha MOS, Toulouse, France) was used to analyze the GAB samples from different fermentation groups. The specific procedure was as follows: 4 mL of the GAB sample was transferred into a 20 mL headspace vial. For headspace extraction, the sample was incubated at 45 °C for 20 min. The injection was performed with the following parameters: continuous injection at 200 °C at a rate of 125 μL·s^−1^ for 25 s. Trapping was conducted at 40 °C in split mode (10 mL·min^−1^) for 30 s. Separation of analytes was achieved through programmed temperature ramping: an initial temperature of 40 °C, ramped at 1 °C·s^−1^ to 120 °C, then at 3 °C·s^−1^ to 250 °C, with a hold time of 60 s. Hydrogen was used as the carrier gas.

### 2.6. GC-IMS Analysis

HS-GC-IMS was used to separate and analyze the VOCs of the four fermentation groups. The HS-GC-IMS method was based on the previously published method [13,18] with minor modifications. The GC-IMS instrument (Fla-vourSpec^®^, G.A.S., Dortmund, Germany) was equipped with a DB-WAX column (30 m × 0.32 mm × 0.25 μm) and an automatic headspace sampler with an injector. First, a two-fold diluted wine sample (4 mL) was placed in a 20 mL sealed headspace vial and incubated at 60 °C for 30 min. Then, 200 μL of the headspace sample (at 85 °C) was injected into the system using a heated syringe, where it was pre-separated by the GC column and coupled with IMS. Ultra-pure nitrogen served as the carrier gas. The GC temperature program was as follows: initial temperature of 40 °C held for 3 min, ramped to 150 °C at 4 °C·min^−1^ and held for 5 min. The pre-separated compounds were ionized in the IMS ionization chamber and then transferred to the drift tube (at 45 °C) with a carrier gas flow of 150 mL·min^−1^. Data processing was conducted using Vocal software (version 0.2.9, G.A.S., Dortmund, Germany). The retention indices (RI) of the volatile compounds were referenced to C4-C9 n-alkanes (Sinopharm Chemical Reagent Co., Ltd., Shanghai, China) and were qualitatively analyzed by comparing RI and drift time (DT) with values in the database.

### 2.7. HS-SPME-GC-MS Analysis

HS-SPME-GC-MS was used to separate and analyze the VOCs of the four fermentation groups. The sample, diluted two-fold and mixed with 2 g of sodium chloride, was placed in a headspace vial with 2-octanol (200 µg·mL^−1^) as the internal standard. The prepared sample was incubated at 55 °C for 15 min, followed by extraction and adsorption using a 50 µm/30 µm CAR/DVB/PDMS SPME fiber for 30 min. The fiber was then immediately inserted into the GC–MS injector for desorption at 250 °C for 0.5 min. The analysis system consisted of a Thermo Trace1310 gas chromatograph ((Thermo Fisher Scientific, Waltham, MA, USA) with a Thermo ISQ LT mass spectrometer and a TG-WAXA column (60 m × 0.25 mm × 0.25 μm). The injector temperature was set to 250 °C, and the GC temperature program was as follows: initial temperature of 40 °C, ramped at 3 °C·min^−1^ to 180 °C, then at 20 °C·min^−1^ to 230 °C, held for 15 min. High-purity helium (99.999%) served as the carrier gas at a flow rate of 1.2 mL·min^−1^. Mass spectrometry conditions were as follows: ion source temperature at 260 °C, electron ionization energy at 70 eV, and monitoring of the total ion current in the mass range of 20–450 m·z^−1^. Retention indices (RIs) were determined using C_6_-C_30_ n-alkane standards. Volatile organic compounds were identified by MS, and by comparing the Kovats retention indices (RI) with the NIST database and literature reports. Quantification of VOCs was based on the internal standard (2-octanol, 200 µg·mL^−1^) and the linear relationship between compound peak area and concentration.

### 2.8. OAV Analysis

The OAV is commonly used to evaluate the contribution of aromatic compounds. Aromatic compounds with OAV > 1 are considered to significantly contribute to the overall aroma characteristics of the GAB. The calculation formula for OAV is as follows: OAVi = Ci/Ti, where Ci and Ti represent the concentration and threshold of the target compound, respectively.

### 2.9. Statistical Analysis

All experiments were conducted with three replicates, and results are expressed as means ± standard deviation (SD). Statistical analysis was performed using one-way ANOVA, followed by Duncan’s post hoc test, with *p* < 0.05 indicating statistical significance. Origin 2021 (Origin Lab Corporation, Northampton, MA, USA) was used to create waterfall plots. The GC-IMS contour and fingerprint analysis were conducted using the gallery plugin. Partial least squares–discriminant analysis (PLS-DA) was performed using Metabo-Analyst 6.0. Use Hiplot to generate a heatmap.

## 3. Results

### 3.1. Analysis of the Quality Parameters of Greengage Alcoholic Beverage

The results of the physicochemical parameters of the GAB are presented in Table 1. SY exhibited the highest sugar utilization rate, with a sugar content of less than 10 g·L^−1^ (8.62 g·L^−1^), complying with the semi-dry fruity alcoholic beverages standard in the Chinese national standard NY/T 1508-2017 [24]. In contrast, the sugar contents in the GAB samples fermented with the other three strains were greater than 12 g·L^−1^ (25.45, 17.63, and 16.65 g·L^−1^), meeting the semi-sweet fruity alcoholic beverages standard. The final alcohol content of the fermented GAB ranged from 10.45% to 11.78% *v*/*v*, with the SY fermentation group achieving the highest alcohol content (11.78% *v*/*v*), which is related to its higher sugar utilization rate. No significant differences in pH were observed among the four fermentation groups. The ET008-c54 fermentation group exhibited the lowest total acid concentration (8.42 g·L^−1^) compared to the other groups. Furthermore, the ET008-c54 fermentation group showed higher color intensity and hue compared to its respective fermentation groups. This study indicates that the color tonality of fermented fruit beverages is closely related to *S. cerevisiae* metabolism [25]. Overall, SY demonstrated superior fermentation performances for GAB brewing.

### 3.2. GC-E-Nose Analysis of Aroma Differences in Greengage Alcoholic Beverage by Different S. cerevisiae

**Figure 1:**
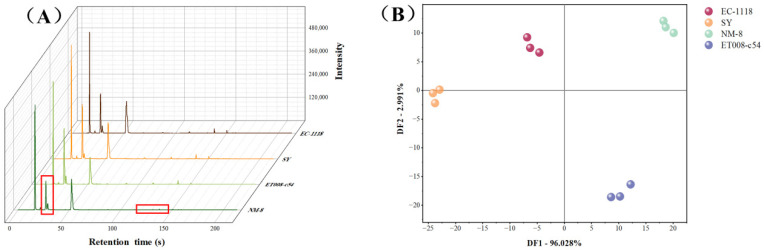
(**A**) Gas chromatography electronic nose (GC-E-Nose) fingerprint profiles of different fermentation groups (MXT-5). The red box in (**A**) represents that the Intensity in the NM-8 fermentation group is significantly lower than that in the other three fermentation groups at 20~30 s and 130~140 s. (**B**) Discriminant function analysis (DFA) results based on GC-E-Nose.

GC-E-Nose is a novel aroma analysis technology that provides a more comprehensive detection of volatile compounds compared to traditional sensor-based electronic noses, offering greater sensitivity and speed [26]. In this study, aroma fingerprint profiles of the GAB were established using signals collected from MXT-5 and MXT-1701 columns. The representative fingerprint profiles of different fermentation groups (MXT-5) are shown in Figure 1A. The results indicated that the flavor compound content in the NM-8 fermentation group is significantly lower than that in the other three fermentation groups at 20~30 s and 130~140 s.

To establish a visual recognition model that distinguishes between different fermentation groups and to analyze the differences among them, discriminant factor analysis (DFA) was performed. The total discriminant factor value of the DFA was 99.019%, which is sufficient to explain the differences among the samples. As shown in Figure 1B, the four fermentation groups were located in four distinct quadrants. The points corresponding to the SY, EC-1118, NM-8, and ET008-c54 fermentation groups fall within the ranges of −24.12~−22.91, −6.79~−4.55, 18.29~20.17 and 8.64~12.24 on DF1, respectively. The distribution of the four fermentation groups along DF1 showed significant differences, particularly between the SY and NM-8 fermentation groups, indicating substantial differences in their over all aroma. The combined results of GC-E-Nose and DFA analysis confirmed that the VOCs of the GAB fermented by the four *S. cerevisiae* strains exhibited obvious differences.

### 3.3. Identification of Volatile Organic Compounds by GC-IMS

#### 3.3.1. Comparison of GC-IMS Topographic Plots

**Figure 2:**
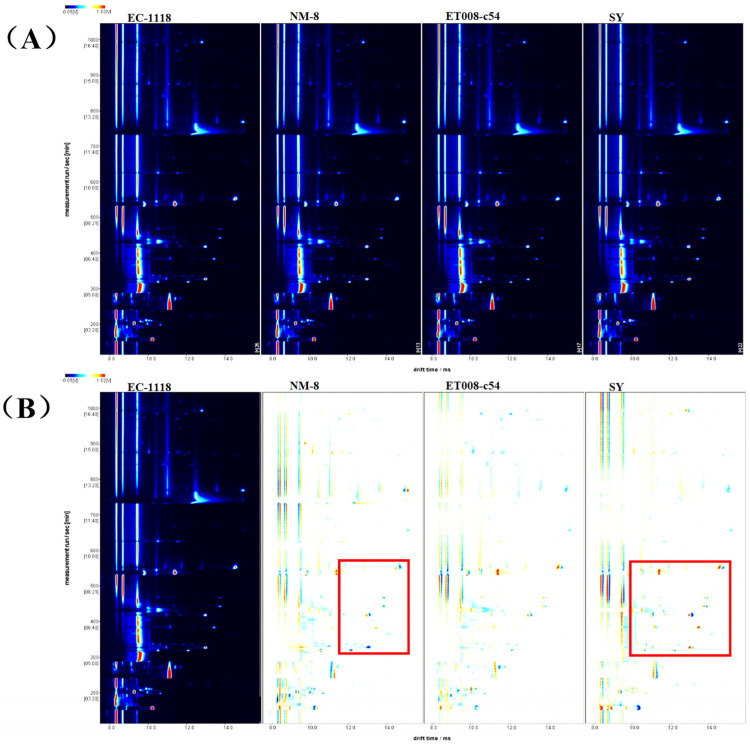
The volatile fingerprints of the different fermentation groups obtained from gas chromatography–ion mobility spectrometry (GC-IMS). (**A**) Topographic plots; (**B**) difference comparison plots. The red box in (**B**) represents the primary regions of the difference comparison plots of the distinct fermentation groups.

As an emerging technique for food flavor analysis, GC-IMS offers significant advantages, such as no sample pre-treatment and rapid analysis, making it highly promising for the detection of trace volatile compounds. To better observe the content differences of VOCs among the various fermentation groups, a two-dimensional spectrum was generated (Figure 2A). In this plot, each bright spot represented a compound, and the color intensity directly reflected the concentration level of volatile compounds: the closer the color was to white, the lower the content; the closer it was to red, the higher the concentration [26,27].

The GC-IMS results showed that the retention time of the volatile components in the GAB mostly ranged from 0 to 1200 s, with a drift time between 1.0 and 1.7 s. To clearly observe intergroup differences, the EC-1118 fermentation group was selected as the reference, and differential spectra were generated by comparing it with other fermentation groups (Figure 2B). Red indicated VOCs higher than the control, while blue indicated lower concentrations, with the intensity representing the degree of difference. Especially in the area marked by the red box, the SY fermentation group displayed the most red points, suggesting a higher concentration of VOCs compared to the other three groups. Notably, NM-8 showed a greater number of dark blue spots, indicating lower VOCs concentrations in these regions compared to the other fermentation groups.

#### 3.3.2. Analysis of GC-IMS Fingerprints

**Figure 3:**
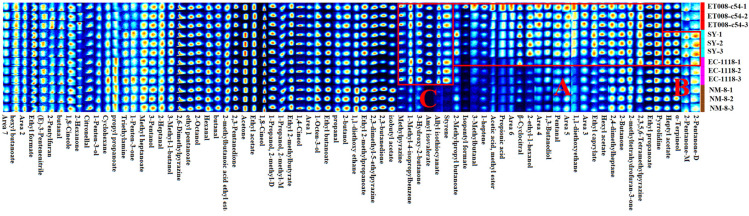
Volatile characteristic fingerprint spectra of the different fermentation groups. The differences among the fermentation groups ET008-c54, SY, EC-1118, and NM-8 were mainly reflected in regions A, B, and C.

HS-GC-IMS was employed to identify and analyze VOCs in the four fermentation groups, resulting in the detection of 75 signal peaks corresponding to 73 VOCs. These included nineteen esters, eleven alcohols, ten ketones, eight aldehydes, two terpenes, one acid, and fourteen other compounds (Table S1). Most of these VOCs were detected predominantly in their monomeric forms. Notably, 1-Propanol, 2-methyl and 2-pentanone were also detected in their dimeric forms, likely due to their concentration or high proton affinity [13]. To facilitate a clearer comparison of VOC differences among the four fermentation groups, a volatile fingerprint spectrum was generated using the Gallery Plot. In this plot, each row represented a sample, while each column corresponded to a VOC. The color intensity reflected the VOCs’ concentration, with brighter colors indicating higher levels. Numbers in the fingerprint indicated compounds with responses unmatched in the spectral library [28]. As shown in Figure 3, the differences among the fermentation groups ET008-c54, SY, EC-1118, and NM-8 were mainly reflected in regions A, B, and C.

In Region A, esters such as ethyl caprylate (apple, pineapple aroma), isopentyl formate (fruity aroma), 2-methylpropyl butanoate (sweet, fruity aroma), and amyl isovalerate (fresh, fruity aroma) were identified. Aldehydes, including β-cyclocitral (rose, herbal aroma), 3-methylbutanal (peach aroma), propanal (nutty aroma), and 2-pentanone (sweet, fruity aroma) were also detected. These aroma compounds were present at significantly higher levels in the SY and ET008-c54 fermentation groups, compared to NM-8 and EC-1118. In Region C, the VOCs in the NM-8 fermentation group were significantly lower than in the other groups, particularly for compounds like amyl isovalerate (fresh apple aroma) and styrene (balsamic, floral aroma). Additionally, Region B showed that α-terpineol (floral aroma) and heptyl acetate (green, fruity aroma) were significantly more abundant in the SY fermentation group compared to others.

The GC-IMS results indicated distinct VOC concentration differences in the GAB fermented by the four *S. cerevisiae* strains, primarily observed in Regions A, B, and C. To further clarify the specific VOC differences among fermentation groups, GC-MS was employed for quantitative analysis of the VOCs, allowing for the identification of key differential metabolites across the groups.

### 3.4. Identification of Volatile Organic Compounds by HS-SPME-GC–MS

#### 3.4.1. Characterization of VOCs in Different Fermentation Groups

**Figure 4:**
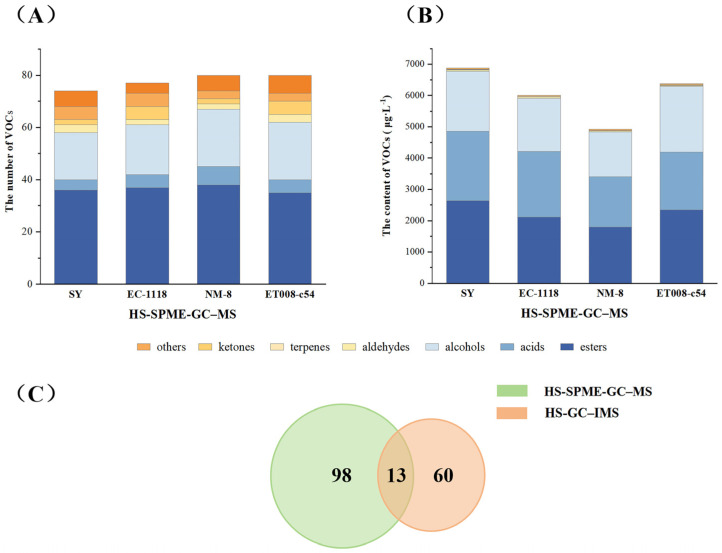
VOC analysis of different fermentation groups by gas chromatography–mass spectrometry (GC-MS): (**A**) comparison of types and quantities of VOCs; (**B**) comparison of VOC contents; (**C**) comparation of the results obtained by HS-GC-IMS and HS-SPME-GC-MS.

A total of one hundred and eleven VOCs were detected by GC-MS, including fifty-two esters, twenty-eight alcohols, eight acids, seven terpenes, five ketones, four aldehydes, and seven other compounds (Table 2, Figure 4A). The fermentation groups SY, EC-1118, NM-8, and ET008-c54 contained 74, 77, 80, and 80 VOCs, respectively. According to the data (Table 2, Figure 4B), the primary VOCs in the GAB were esters (1796.53–2640.94 μg·L^−1^), acids (1612.94–2214.16 μg·L^−1^), and alcohols (1420.56–2094.17 μg·L^−1^).

During fermented fruit alcoholic beverage fermentation, esters were mainly formed through esterification reactions between alcohols and acids [29]. Although there was no significant difference in the number of ester compounds across the fermentation groups, there was substantial variation in their concentrations (Figure 4B). The ester content in the SY, ET008-c54, EC-1118, and NM-8 fermentation groups was 2640.94 μg/L^-1^, 2345.69 μg·L^-1^, 2120.21 μg·L^-1^, and 1796.53 μg·L^-1^, respectively, accounting for 35.30%–38.40% of the total VOCs. Among the esters, ethyl esters were predominant, constituting 95.20%–96.31% of the ester compounds. Compounds such as ethyl octanoate (apricot, banana aroma), ethyl laurate (floral, green apple aroma), and ethyl hexanoate (banana aroma) were present in significantly higher concentrations in the SY fermentation group than in the other groups. This likely contributed to a more pronounced fruity and floral aroma in the GAB fermented with SY, compared to the samples fermented with the other three *S. cerevisiae* strains.

Higher alcohols were produced by *S. cerevisiae* through the Ehrlich pathway by degrading amino acids [20]. Arcena M. R. et al. [25] indicated that excessive levels of higher alcohols can lead to an unpleasant taste, while insufficient levels may result in a thin body. The alcohol content in the ET008-c54 and SY fermentation groups (2094.17 μg·L^-1^, 1914.73 μg·L^-1^) was significantly higher than in the EC-1118 and NM-8 groups (1694.63 μg·L^-1^, 1420.56 μg·L^-1^). Isoamyl alcohol (alcohol, cheese, floral aroma) accounted for 55.84% to 62.66% of the total alcohol content, making it the primary alcohol component in the GAB, consistent with the findings of Tian et al. [30]. Additionally, phenylethyl alcohol and isobutanol were also major alcohols in the GAB, contributing floral, fruity, and green aromas to the fermented alcoholic beverage.

Most of the acid compounds were derived from greengage itself, with a few generated during fermentation [20]. Although the variety of acids in the GAB was limit, the concentrations of acids accounted for 29.03% to 34.92% of the total VOCs, second only to esters. Decanoic acid (803.28–1189.8 μg·L^-1^), octanoic acid (659.3–901.38 μg·L^-1^), and lauric acid (57.27–106.43 μg·L^-1^) were present in relatively high concentrations. Decanoic acid has herbal aromas, octanoic acid produces cheese and fatty aromas, and lauric acid imparts fruity and fatty aromas. The contents of all above three acid compounds were all the most abundant in the SY fermentation group.

Aldehydes were mainly formed through lipid oxidation and amino acid degradation, then quickly converted into acids, resulting in their low concentration. Due to their low odor thresholds, aldehydes can still significantly impact fermented alcoholic beverage flavor even at low concentrations [10]. For instance, citronellal imparted green and citrus aromas, while benzaldehyde contributed sweetness and floral aromas. Terpenes were partly derived from the fruit and exist in both free and glycosidically bound forms [31], with others being produced through the MVA pathway of *S. cerevisiae* strains, commonly in monoterpene form [32]. Although terpene compounds were low in total concentration (14.5–18.42 μg·L^-1^), they played an essential role in enhancing the fermented fruit alcoholic beverage aroma with their unique floral characteristics [33,34].

Based on the identification results from GC-MS and GC-IMS, a total of 171 VOCs were detected in the GAB, as shown in Figure 4C. Comparing the two detection methods, 98 VOCs were detected only by GC-MS, 60 VOCs were detected only by GC-IMS, and 13 VOCs were detected by both. GC-MS detected a larger number of VOCs, indicating its potential to provide a broader range of odor information for the GAB. However, certain trace aldehydes, such as hexanal, propanal, and β-cyclocitral, were detectable only by GC-IMS, demonstrating that GC-IMS has higher sensitivity for aldehyde recognition. Therefore, combining these two analytical methods enabled a more comprehensive investigation of VOCs in the GAB.

#### 3.4.2. Multivariate Statistical Analysis

**Figure 5:**
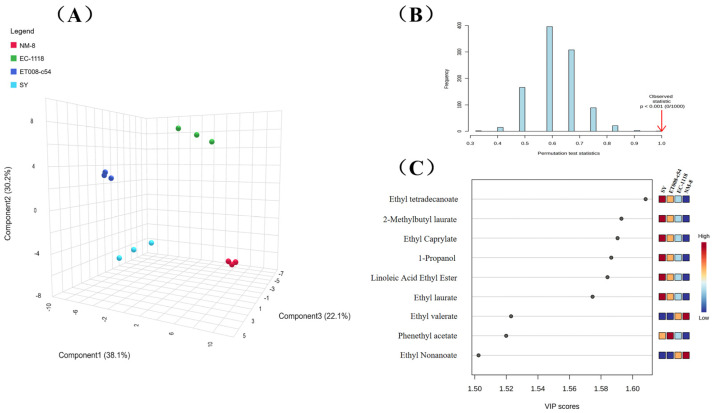
(**A**) Partial least squares–discriminant analysis (PLS-DA) of GC-MS data; (**B**) permutation test statistics of PLS-DA; (**C**) variable importance in projection (VIP) scores of each variable.

To gain deeper insights into the differences in VOCs among the GAB fermentation groups, PLS-DA analysis was applied to the GC-MS results. PLS-DA is a supervised discriminant analysis method that allows for visualization of similarities and differences between samples [11]. The greater the difference between the two samples, the further apart they appear on the score plot [35]. The overall discriminant effect of the PLS-DA model is shown in Figure 5A. In the 3D score plot, Component 1 (38.1%), Component 2 (30.2%), and Component 3 (22.1%) together explained 90.4% of the total variance, indicating that the score plot effectively captured the similarities or differences between samples. The statistical validation parameters of model (R^2^ = 0.99, Q^2^ = 0.97) indicated high accuracy and reliability. And the results of 1000 permutation tests confirmed there was no model overfitting (Figure 5B). The PLS-DA model showed clear separation among the four fermentation groups, indicating significant differences in VOC profiles among the GAB fermented by the four *S. cerevisiae* strains, consistent with the GC-E-Nose and GC-IMS results. 

Variable importance in projection (VIP) scores were used to assess the influence and explanatory power of each variable for classifying and discriminating between groups. Higher VIP values indicated greater differences in aroma compounds between groups. Using VIP analysis, the key VOCs distinguishing the four fermentation groups were identified. By setting VIP > 1.5 as the selection criterion and combining it with one-way ANOVA (*p* < 0.05), nine volatile markers were identified (Figure 5C). These markers included ethyl myristate (VIP = 1.61), methyl laurate (VIP = 1.59), ethyl octanoate (VIP = 1.59), 1-propanol (VIP = 1.59), ethyl linoleate (VIP = 1.58), ethyl laurate (VIP = 1.57), ethyl valerate (VIP = 1.52), phenethyl acetate (VIP = 1.52), and ethyl nonanoate (VIP = 1.51). The heatmap of the VIP score plot indicated the different concentrations of these compounds across the fermentation groups. Among VOCs with VIP > 1.5, ethyl esters were dominant, underscoring their critical role in differentiating the GAB fermented by various *S. cerevisiae* strains. The total content of ethyl esters with VIP > 1.5 was 1040.48 μg·L^-1^, 824.58 μg·L^-1^, 731.44 μg·L^-1^, and 619.33 μg·L^-1^ for the SY, ET008-c54, EC-1118, and NM-8 fermentation groups, respectively. Ethyl myristate (pleasant aroma), ethyl octanoate (fresh, banana aroma), ethyl linoleate (fruity aroma), and ethyl laurate (green apple aroma) showed decreasing concentrations in the SY, ET008-c54, EC-1118, and NM-8 groups, respectively. Additionally, phenethyl acetate (rose aroma) had the highest concentration in NM-8, while ethyl valerate (apple, sweet aroma) and ethyl nonanoate (banana, grape aroma) were detected only in the ET008-c54 and EC-1118 fermentation groups.

#### 3.4.3. Key Volatile Components of Greengage Alcoholic Beverage by OAV Analysis

The contribution of VOCs to the overall aroma of the GAB depended on their OAV, which was influenced by both their concentration and odor threshold [36]. Based on the concentration and odor threshold of the VOCs, a total of 13 aroma compounds with OAV ≥ 1 were identified, including seven esters, two alcohols, two ketones, one acid, and one olefin.

Among these compounds, combined with the VIP values, ethyl octanoate (OAV = 69.67–105.85) emerged not only as a potential marker for distinguishing aroma differences among the GAB samples fermented by different *S. cerevisiae* strains, but also as a key contributor to the characteristic aroma of the GAB. Notably, β-Ionone exhibited an exceptionally high OAV (OAV = 982.86–1107.14), largely due to its low odor threshold (0.007 μg/L), making it a prominent contributor to the overall flavor of the GAB. In previous research, β-Ionone has been identified as a characteristic aroma compound in green tea beverages and citrus tea, offering a violet-like aroma [36,37]. Noguerol-Pato et al. highlighted β-Ionone as a unique aroma volatile in red wine [38]. Additionally, ethyl laurate was present above its threshold (OAV = 1.06) only in the SY fermentation group, which contributes floral and green apple aromas to the beverage as previously mentioned. Ethyl isobutyrate (OAV = 6.7–20.8) and ethyl 2-methylbutyrate (OAV = 6.05–8.7) were also major contributors to the GAB aroma, consistent with the findings by Tian et al [30].

**Figure 6:**
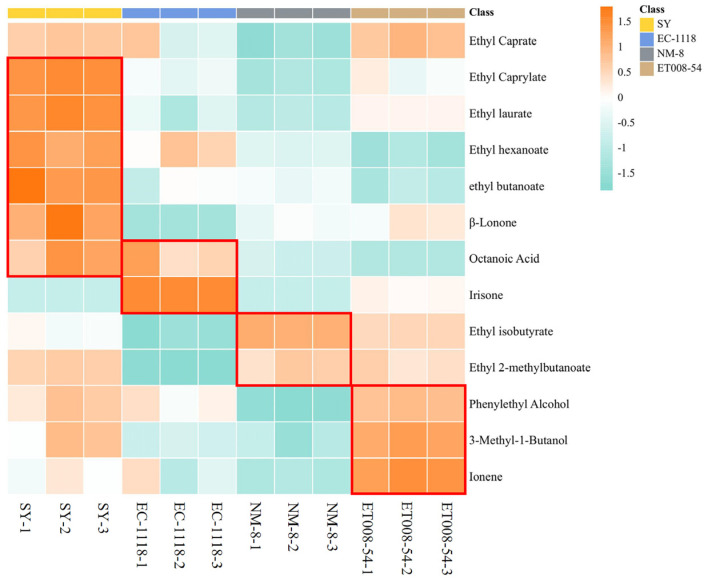
The heatmap of aroma compounds in fermentation groups from four *S. cerevisiae* strains. The red box represents the differences of compounds with OAV ≥ 1 among different fermentation groups (ET008-c54, SY, EC-1118, and NM-8).

To visualize the contribution of each VOC to the aroma in the different fermentation groups, a heatmap was generated (Figure 6) based on one-way ANOVA (*p* < 0.05) for compounds with OAV ≥ 1. The results showed that in the SY fermentation group, β-Ionone, ethyl octanoate (fresh, fruity aroma), ethyl laurate, ethyl hexanoate (banana, cheese aroma), ethyl butyrate (apple, butter aroma), and octanoic acid (acidic, cheese, fatty aroma) made significant aroma contributions. In the EC-1118 group, irisone (floral aroma) was a prominent contributor. For the NM-8 group, ethyl isobutyrate (floral, fruity aroma) showed a relatively high aroma contribution, though the overall aroma was less pronounced. In the ET008-54 group, isopentanol (alcohol, cocoa, floral aroma), ionene (floral aroma), and ethyl decanoate (fruity, brandy-like aroma) dominated the aroma profile. Overall, the SY fermentation group produced the richest aroma, followed by ET008-54, and EC-1118, with the NM-8 group showing a comparatively mild aroma profile.

### 3.5. Sensory Evaluation

**Figure 7:**
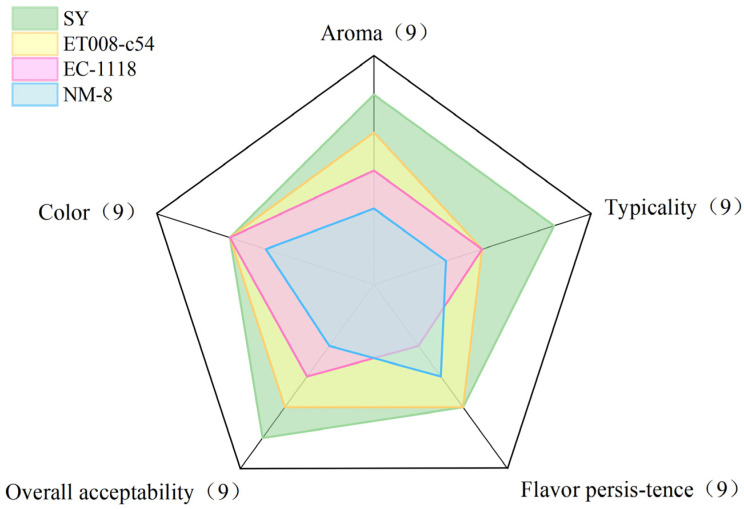
Radar chart of scores for the sensory evaluation of the GAB fermented by four *S. cerevisiae* strains. Each attribute was scored on a scale from 0 to 9, where 0 indicated extremely weak and 9 indicated extremely strong.

Results from the sensory evaluation (Figure 7) indicated that the GAB fermented by SY received the highest overall score, while NM-8 scored the lowest. Additionally, the SY fermentation group scored higher in aroma, typicality, and overall acceptability compared to the other groups, likely due to SY producing a greater quantity of aromatic VOCs during GAB fermentation, enhancing the distinctive aroma of the GAB. In terms of flavor persistence, EC-1118 performed less satisfactorily. In addition, there was no significant difference in color among the four fermentation groups.

## 4. Conclusions

*S. cerevisiae* was a critical factor influencing the flavor quality of the fermented fruit alcoholic beverage. This study used GC-E-Nose, GC-MS, and GC-IMS techniques to characterize the VOCs in the GAB fermented by four different *S. cerevisiae* strains. Combining these three methods compensated for their limitations, providing a more comprehensive understanding of VOC differences across fermentation groups. GC-E-Nose, combined with DFA, showed significant flavor differences in the GAB samples fermented by the four strains. A total of 171 VOCs were identified using GC-MS and GC-IMS, with 98 VOCs detected only by GC-MS, 60 exclusively by GC-IMS, and 13 by both methods. GC-MS identified more VOCs, supplying extensive flavor information for the GAB, while GC-IMS was more sensitive in detecting trace compounds, especially aldehydes like hexanal, propanal, and β-cyclocitral, which were identified exclusively by GC-IMS.

Using PLS-DA and VIP values, nine aroma compounds (VIP > 1.5) were selected as potential markers, with ethyl esters (such as ethyl myristate, 2-lauric acid methyl ester, ethyl octanoate, ethyl linoleate, ethyl laurate, ethyl valerate, phenylethyl acetate, and ethyl nonanoate) playing a dominant role in differentiating aroma profiles among the fermentation groups. Additionally, OAV analysis identified 13 aroma compounds with OAV ≥ 1, including β-ionone and ethyl octanoate as representative aroma compounds of the GAB, both of which contributed to a strong floral and fruity aroma and were most abundant in the SY fermentation group. Notably, ethyl octanoate emerged as both a potential marker for distinguishing aroma variations in the GAB fermented by different *S. cerevisiae* strains and as an important contributor to the overall aroma of the GAB.

Combined with the sensory evaluation, the analysis indicated that the SY fermentation group exhibited a more distinctive flavor profile. These findings provided a useful basis for studying aroma differences in the GAB fermented by different *S. cerevisiae* strains and offered theoretical guidance for selecting *S. cerevisiae* strains in the industrial production of the GAB.

## Figures and Tables

**Table 1:** Results of physiochemical characteristics of GAB fermented by different *Saccharomyces cerevisiae* strains.

Physicochemical Parameters	SY	ET008-c54	EC-1118	NM-8
Reducing sugar (g·L^−1^)	8.62 ± 0.59 a	16.65 ± 1.12 b	25.45 ± 1.11 c	17.63 ± 0.63 b
Sugar utilization (%)	0.87 ± 0.01 d	0.83 ± 0.01 b	0.78 ± 0 a	0.77 ± 0.01 a
Total acid (g·L^−1^)	10.04 ± 0.11 ab	8.42 ± 1.95 a	10.3 ± 0.23 ab	10.56 ± 0.26 b
Alcohol degree (%*v*/*v*)	11.78 ± 0.08 c	11.17 ± 0.07 b	10.53 ± 0.05 a	10.45 ± 0.1 a
pH	2.45 ± 0.02 b	2.44 ± 0.01 ab	2.41 ± 0.02 a	2.45 ± 0.02 b
Color intensity	0.61 ± 0.01 b	0.67 ± 0.02 c	0.56 ± 0.01 a	0.57 ± 0.01 a
Color tonality	2.43 ± 0.11 b	2.97 ± 0.07 c	1.93 ± 0.03 a	1.97 ± 0.08 a

Color intensity = A420 + A520 + A620. Color tonality = A420/A520. Values with different letters (a–d) in the same row indicated significant differences (*p* < 0.05).

**Table 2:** Volatile components determined by GC-MS in the GAB fermented by different *Saccharomyces cerevisiae* strains.

Compound	RI ^δ^	Identification ^ε^	Concentration (μg·L^−1^) ^η^				Threshold (μg·L^−1^) ^θ^	Aroma Description ^θ^
			SY	EC-1118	NM-8	ET008-c54		
Ethyl Caprate	1629	MS, RI	841.89 ± 8.95 a	744.54 ± 88.57 a	581.76 ± 14.49 b	858.98 ± 15.46 a	200	brandy, fruit, grape
Ethyl Caprylate	1426	MS, RI	529.24 ± 3.3 a	412.26 ± 8.44 b	348.37 ± 4.83 c	426.17 ± 18.94 b	5	banana, fresh, fruit
Ethyl Laurate	1831	MS, RI	425.85 ± 9.36 a	259.86 ± 37.48 c	231.07 ± 3.57c	319.29 ± 0.68 b	400	cream, floral, green apple,
Ethyl Acetate	850	MS, RI	226.37 ± 2.69 a	145.13 ± 4.77 c	221.09 ± 1.62 a	205.25 ± 3.38 b	750	fruit, grape, pineapple
Ethyl Palmitate	2235	MS, RI	125.85 ± 11.65 a	122.6 ± 1.51 ab	94.23 ± 18.54 b	120.21 ± 6.48 ab	2000	fat, fruit, sweet
Ethyl Hexanoate	1223	MS, RI	130.47 ± 3.87 a	113.3 ± 8.39 b	93.56 ± 0.43 c	76.43 ± 3.62 d	14	apple, banana, cheese
Ethyl Benzoate	1659	MS, RI	114.19 ± 1.81 a	112.73 ± 8.01 a	70.71 ± 4.25 c	96.68 ± 2.8 b	740	fat, floral, flower
Ethyl 9-Hexadecenoate	2262	MS, RI	26.6 ± 7.95 a	25.36 ± 0.96 a	ND	28.71 ± 0.05 a	NF	NF
Phenethyl Acetate	1804	MS, RI	40.12 ± 1.52 b	25.17 ± 1.97 c	15.57 ± 0.46 d	43.78 ± 0.21 a	650	floral, fruit, honey, rose
Ethyl Tetradecanoate	2040	MS, RI	25.57 ± 0.14 a	16.89 ± 0.49 b	11.09 ± 1.04 c	19.18 ± 0.98 d	180	cream, oil, pleasant
Isoamyl Acetate	1110	MS, RI	29.05 ± 2.29 a	19.36 ± 1.84 b	28.23 ± 0.57 a	27.86 ± 1.37 a	30	apple, banana, fruit
isoamyl Decanoate	1850	MS, RI	20.09 ± 0.26 a	18.8 ± 3.16 a	8.03 ± 0.2 b	19.14 ± 0.17 a	5000	wax
Ethyl Stearate	2439	MS, RI	13 ± 1.08 b	10.21 ± 0.69 c	7.46 ± 1.32 d	20.34 ± 0.18 a	500	wax
2-Methylbutyl Laurate	2059	MS, RI	9.94 ± 1.43 a	5.55 ± 0.79 b	2.91 ± 0.16 c	6.8 ± 0 b	450	NF
Linoleic Acid Ethyl Ester	2511	MS, RI	9.76 ± 0.61 a	6.54 ± 0.35 b	4.69 ± 0.13 c	9.36 ± 0.04 a	450	fruit
3-methylbutyl Octanoate	1648	MS, RI	9.07 ± 0.09 a	10.44 ± 1.16 a	5.34 ± 0.14 b	9.71 ± 0.06 a	70	baked apple
2,2,4-Trimethyl-1,3-Pentanediol Diisobutyrate	1864	MS, RI	2.9 ± 0.42 a	ND	2.17 ± 0.02 b	ND	NF	NF
Ethyl Butanoate	1022	MS, RI	7.59 ± 0.3 a	5.59 ± 0.54 bc	5.69 ± 0.1 b	4.78 ± 0.19 c	0.9	apple, banana, fruit
Ethyl Phenylacetate	1774	MS, RI	8.31 ± 0.29 a	6.88 ± 0.44 c	7.3 ± 0.27 bc	7.89 ± 0.1 ab	250	honey, rose, sweet
R-γ-Decalactone	2138	MS, RI	6.48 ± 0.15 a	6.41 ± 0.14 a	5.6 ± 0.5 b	6.18 ± 0.11 ab	70	fruit, peach, pleasant
Isobutyl Decanoate	1744	MS, RI	ND	ND	ND	5.16 ± 0.03 a	NF	baked apple
Ethyl 9-Decenoate	1679	MS, RI	ND	8.83 ± 0.63 b	4.05 ± 0.14c	5.28 ± 0.28 d	NF	NF
Isopropyl Palmitate	2221	MS, RI	4.93 ± 1.24 a	5.16 ± 1.66 a	4.33 ± 1.07 a	5.13 ± 0.1 a	NF	NF
Benzylcarbinyl Caproate	2157	MS, RI	ND	4.19 ± 0.02 a	ND	3.74 ± 0.08 b	94	NF
Dibutyl Phthalate	2684	MS, RI	5.33 ± 0.83 ab	5.88 ± 0.94 ab	7.81 ± 1.94 a	4.62 ± 0.03 b	260	NF
2-Phenylethyl Pentanoate	2372	MS, RI	4.45 ± 0.02 a	ND	ND	ND	NF	NF
Ethyl Linolenate	2578	MS, RI	2.92 ± 0.23 a	1.88 ± 0.36 b	ND	2.89 ± 0.09 a	NF	NF
Propanoic Acid, Ethyl Ester	935	MS, RI	2.14 ± 0.14 a	1.3 ± 0.1 c	1.46 ± 0.01 c	1.85 ± 0.02 b	10	apple, fruit, pineapple
Ethyl Isovalerate	1051	MS, RI	1.08 ± 0.01 b	ND	2.32 ± 0.3 a	ND	3	apple, citrus, fruit
Ethyl Isobutyrate	944	MS, RI	1.69 ± 0.08 c	0.67 ± 0.09 d	2.46 ± 0.02 a	2.08 ± 0.01 b	0.1	apple, floral, fruit,
Ethyl 2-Methylbutanoate	1036	MS, RI	1.74 ± 0.01 a	1.21 ± 0 b	1.72 ± 0.04 a	1.69 ± 0.04 a	0.2	apple, floral, fruit
Ethyl Margarate	2337	MS, RI	ND	0.64 ± 0.01 a	ND	ND	NF	NF
Phenethyl Decanoate	2589	MS, RI	ND	ND	ND	1.06 ± 0 a	NF	NF
Propyl Caprate	1712	MS, RI	1.17 ± 0 ab	1 ± 0.11 a	ND	1.21 ± 0.08 b	NF	fruit
Hexyl Formate	1339	MS, RI	0.73 ± 0.02 b	0.97 ± 0.05 a	1.01 ± 0.06 a	ND	NF	NF
Diethyl Bis(trimethylsilyl) Silicate	1169	MS, RI	0.36 ± 0.04 a	0.26 ± 0 b	0.31 ± 0.02 ab	0.33 ± 0.01 a	NF	NF
2-Ethylhexyl Acetate	1374	MS, RI	ND	0.15 ± 0.05 b	ND	0.72 ± 0.03 a	43	NF
Octyl Formate	1542	MS, RI	ND	ND	5.62 ± 0.55 a	ND	NF	NF
Phenethyl Nonanoate	2157	MS, RI	4.45 ± 0.05 a	ND	ND	ND	NF	NF
Ethyl 3-Phenylpropanoate	1873	MS, RI	2.44 ± 0.18 b	ND	2.98 ± 0.12 a	2.06 ± 0.07 c	14	floral, fruit, honey
Ethyl Valerate	1122	MS, RI	ND	0.21 ± 0 b	0.25 ± 0.01 a	ND	1.5	apple, herb, sweet
Phenethyl Butyrate	1869	MS, RI	ND	3.03 ± 0.03 a	ND	ND	961	fruit
Isobutyl Acetate	1000	MS, RI	ND	ND	0.69 ± 0 b	0.72 ± 0 a	25	apple, floral, herb
Diisobutyl phthalate	2529	MS, RI	3.27 ± 0.69 a	ND	4.24 ± 0.74 a	ND	NF	NF
Benzyl Acetate	1718	MS, RI	ND	ND	1.17 ± 0.03 a	ND	364	fresh, fruit, honey
Ethyl Hex-3-enoate	1292	MS, RI	ND	ND	0.36 ± 0 a	ND	NF	NF
Ethyl (Z)-3-Hexenoate	1291	MS, RI	0.35 ± 0 a	ND	ND	ND	10	fruit
2-Phenylethyl Dodecanoate	2588	MS, RI	1.55 ± 0.01 a	ND	ND	ND	NF	NF
Decyl Decanoate	1744	MS, RI	ND	6.89 ± 0.7 a	5.07 ± 0.01 b	ND	NF	NF
Octyl Octanoate	1542	MS, RI	ND	5.36 ± 0.02 a	ND	ND	NF	NF
Ethyl Nonanoate	1525	MS, RI	ND	4.96 ± 0.07 a	5.38 ± 0 a	ND	377	banana, fruit, grape
Ethyl Heptanoate	1322	MS, RI	ND	ND	0.43 ± 0.01 a	0.41 ± 0 b	18	brandy, fruit, wine
3-Methyl-1-Butanol	1193	MS, RI	1109.8 ± 64.62 a	946.3 ± 11.92 c	890.19 ± 45.55 c	1197.4 ± 12.09 a	600	banana, cheese, floral
Phenylethyl Alcohol	1894	MS, RI	678.27 ± 40.71 ab	617.67 ± 36.58 b	367.15 ± 7.5 c	714.4 ± 6.66 a	564.23	floral, fruit, honey, rose
Isobutanol	1076	MS, RI	40.73 ± 2.57b	49.65 ± 3.25 b	67.85 ± 6.02 a	61.82 ± 1.23 a	25,000	alcohol, apple
2-Ethylhexanol	1474	MS, RI	16.85 ± 0.52d	23.16 ± 0.24 c	24.92 ± 0.16b	44.12 ± 0.92 a	300	citrus, green, oil, rose
Nerolidol	2025	MS, RI	13.37 ± 0.96 a	10.22 ± 1.24 b	5.68 ± 0.12 c	13.82 ± 0.28 a	250	oil, flower, wood
Benzyl Alcohol	1860	MS, RI	12.44 ± 0.81b	10.56 ± 0.71 c	15.69 ± 0.33 a	12.11 ± 0.27 b	159,000	floral, fruit
1-Propanol	1019	MS, RI	8.68 ± 0.67 a	5.87 ± 0.38 b	4.99 ± 0.53b	7.62 ± 0.19 a	25,000	alcohol, candy, plastic
1-Dodecanol	1948	MS, RI	6.58 ± 1.79 a	4.78 ± 1.36 a	5.13 ± 0.35 a	5.7 ± 0.57 a	1000	fat, wax
1-Octanol	1542	MS, RI	4.58 ± 0.06 a	ND	5.27 ± 0.68 a	ND	820	citrus, fat, fruit, green
Dehydrolinalool	1595	MS, RI	5.03 ± 0.26 b	4.64 ± 0.11 b	5.63 ± 0.12 a	4.89 ± 0.09 b	NF	fresh, lemon, sweet
(R)-(+)-citronellol	1748	MS, RI	ND	ND	ND	1.95 ± 0.49 a	40	floral
1-Nonanol	1643	MS, RI	1.85 ± 0.09 b	2.05 ± 0.06 ab	1.68 ± 0.14 b	2.35 ± 0.31 a	310	fat, floral, green
Linalool	1533	MS, RI	1.09 ± 0.24 b	1.05 ± 0.12 b	ND	2.23 ± 0.07 a	6	floral, flower, grape
1-Hexadecanol	2351	MS, RI	ND	2.97 ± 0.61 a	3.98 ± 0.94 a	3.93 ± 0.62 a	1100	flower, wax
Cis-Ocimenol	1638	MS, RI	2.39 ± 0.05 b	2.07 ± 0.07 c	ND	3.15 ± 0.15 a	NF	NF
1-Butanol	1129	MS, RI	1.11 ± 0.08 ab	1.07 ± 0.08 b	1.29 ± 0.09 a	0.73 ± 0.02 c	459.2	alcohol, fermented, fruit
1-Octen-3-Ol	1436	MS, RI	0.83 ± 0.01 a	ND	0.77 ± 0.03 c	0.64 ± 0 d	20	floral, green, herb
Farnesol	2328	MS, RI	2.59 ± 0.03 a	2.81 ± 0.76 a	1.03 ± 0.03 b	2.82 ± 0.1 a	20	floral, oil, weet
1-Octadecanol	2351	MS, RI	2.49 ± 0.01 a	ND	1.06 ± 0.01 b	ND	NF	oil
(E)-2,6-Dimethyl-5,7-Octadien-2-Ol	1638	MS, RI	ND	ND	2.78 ± 0.04 a	ND	NF	NF
Methionol	1700	MS, RI	ND	ND	2.65 ± 0.01 b	2.88 ± 0.14 a	36	cooked potato, earth
(Z)-Linalool Oxide (Furanoid)	1431	MS, RI	ND	ND	1.81 ± 0.04 b	1.88 ± 0.27 b	100	earth, flower,
1-Hexanol	1338	MS, RI	ND	0.9 ± 0.03 a	ND	0.76 ± 0.06 b	8000	flower, fruit, green
(3S)-3,7-Dimethyloct-7-en-1-ol	1748	MS, RI	ND	2.55 ± 0.03 a	ND	ND	NF	floral
1-Decanol	1745	MS, RI	ND	ND	5.5 ± 0.1 a	ND	500	fat, oil, plastic
α-Bisabolol	2198	MS, RI	ND	ND	ND	3.4 ± 0.08 a	NF	flower, spice
Cis-3-Nonen-1-Ol	1667	MS, RI	ND	0.64 ± 0.01 a	0.43 ± 0.04 b	ND	NF	wax
α-Terpineol	1684	MS, RI	6.05 ± 0.21 a	5.67 ± 0.37 ab	5.08 ± 0 b	5.57 ± 0.27 ab	250	floral, fresh, oil
N-Decanoic Acid	2247	MS, RI	1189.8 ± 74.33 a	1145.04 ± 69.16 a	803.28 ± 29.9 b	1094.41 ± 20.65 a	9400	fat, grass, rancid
Octanoic Acid	2046	MS, RI	901.38 ± 45.13 a	865.57 ± 48.74 a	707.06 ± 6.93 b	659.3 ± 0 b	500	acid, cheese, fat
Dodecanoic Acid	2470	MS, RI	106.43 ± 10.99 a	69.26 ± 8.98 b	57.27 ± 5.73 b	71.49 ± 2.15 b	7200	fat, fruit, metal, wax
Acetic Acid	1452	MS, RI	16.55 ± 2.85 c	10.37 ± 0.87 d	28.41 ± 0.05 a	23.21 ± 1.31 b	26,000	acid, fruit, pungent
9-Decenoic Acid	2310	MS, RI	ND	6.98 ± 0.07 a	ND	ND	4300	soap
Benzoic Acid	2448	MS, RI	ND	ND	3.14 ± 0.07 a	ND	1000	balsamic
Nonanoic Acid	2144	MS, RI	ND	ND	11.19 ± 4.91 a	4.15 ± 0.14 b	1100	cheese
4-Hexyl-2,5-Dioxofuran-3-Acetic Acid	2098	MS, RI	ND	ND	2.59 ± 0.3 a	ND	NF	NF
β-Lonone	1930	MS, RI	7.75 ± 0.29 a	5.78 ± 0.01 c	6.61 ± 0.11 b	6.88 ± 0.18 b	0.007	floral, raspberry, seaweed
2-Octanone	1278	MS, RI	ND	2.06 ± 0.25 a	ND	2.08 ± 0.07 a	50.2	fat, fragant, green
2-Nonanone	1382	MS, RI	ND	0.68 ± 0.02 a	ND	0.5 ± 0.07 b	10.9	fragant, fruit, green
2,6-Di-Tert-Butyl-4-Hydroxy-4-Methylcyclohexa-2,5-Dien-1-One	2082	MS, RI	1.54 ± 0.44 a	1.21 ± 0.01 a	1.69 ± 0.02 a	1.75 ± 0.13 a	NF	NF
Irisone	1929	MS, RI	ND	6.73 ± 0.01 a	ND	2.68 ± 0.15 b	0.45	floral, sweet, violet
3,4-Dimethylbenzaldehyde	1814	MS, RI	32.4 ± 1.04 a	28.5 ± 2.77 ab	26.2 ± 1.58 b	ND	NF	NF
Benzaldehyde	1523	MS, RI	9.79 ± 1.25 a	10.62 ± 0.5 a	9.32 ± 0.32 a	11.2 ± 0.65 a	350	almond, berry, bitter
Isovaleraldehyde	896	MS, RI	0.33 ± 0.01 b	ND	ND	0.43 ± 0.04 a	4.6	chocolate, corn flakes
2,5-Dimethylbenzaldehyde	1814	MS, RI	ND	ND	ND	12.79 ± 1.08 a	200	NF
2,4-Di-Tert-Butylphenol	2282	MS, RI	20.88 ± 0.38 a	19.22 ± 1.92 ab	18.05 ± 1 ab	16.55 ± 1.98 b	500	NF
Eugenol	2152	MS, RI	9.2 ± 0.57 a	ND	9.68 ± 0.15 a	8.56 ± 0.86 a	10	burnt, clove, smoke
4-tert-Amylphenol	2389	MS, RI	2.77 ± 0.06 a	ND	2.22 ± 0.07 b	2.25 ± 0.1 b	800	NF
3-Ethoxy-1-propanol	1362	MS, RI	5.07 ± 0.17 a	4.5 ± 0.18 b	ND	4.68 ± 0.32 ab	100	fruit
Nerol Oxide	1462	MS, RI	3.54 ± 0.01 a	2.77 ± 0.01 b	3.35 ± 0.2 a	3.56 ± 0.14 a	80	flower, oil
Geranic Oxide	1095	MS, RI	1.9 ± 0.06 b	1.84 ± 0.29 b	2.26 ± 0.08 ab	2.41 ± 0.12 a	NF	NF
Ocimene Quintoxide	1233	MS, RI	ND	ND	1.1 ± 0.06 a	0.91 ± 0.08 b	NF	Citrus, wood

^δ^ The retention index of aroma components on DB-Wax; ^ε^ identification based on MS (mass spectrum) and RI (Kovats retention index). ^η^ “ND” =not detected; values with different letters (a–d) in a row were significantly different using Duncan’s multiple comparison tests (*p* < 0.05). **^θ^** Odor description and threshold of all volatiles found in the literature and website: “compilations of odour threshold values in air, water and other media”; https://www.vcf-online.nl/OFTVCompoundSearch.cfm (accessed on 30 October 2024); “NF”: not found.

## Data Availability

The original contributions presented in the study are included in the article/Supplementary Material; further inquiries can be directed to the corresponding authors.

## Supplementary Materials

The following supporting information can be downloaded at: https://www.mdpi.com/article/10.3390/foods13243984/s1, Table S1: Volatile components determined by GC-IMS in GAB fermented by different *Saccharomyces cerevisiae* strains; Table S2: Presents the odor activity values (OAV ≥ 1) for GAB fermented by different *Saccharomyces cerevisiae* strains.
