# Supplementary material for: Exploring the Impact of Different Saccharomyces cerevisiae Strains on the Flavor Profile of Greengage Alcoholic Beverage Using GC-E-Nose, HS-GC-IMS, and HS-SPME-GC-MS
Source: Foods. 2024 Dec 10;13(24):3984. doi: 10.3390/foods13243984 (PMC11675471; doi:10.3390/foods13243984)
Supplement: Supplementary file 1 [file foods-13-03984-s001.zip › foods-3317396-supplementary.pdf]

Table S1. Volatile components determined by GC-IMS in GAB fermented by different *Saccharomyces cerevisiae* strains

Compound	RI	Rt [sec]	Dt [a.u.]	CAS	Peak height			
					ET008-c54	SY	EC-1118	NM-8
β-Cyclocitral	1618.6	1785.288	1.31396	432-25-7	0.13±0.02a	0.16±0.01a	0.14±0.01a	0.14±0.02a
2,3,5,6-Tetramethylpyrazine	1481.9	1381.052	1.21821	1124-11-4	0.2±0ab	0.17±0.03ab	0.15±0.04b	0.22±0a
Citronellal	1478.9	1373.261	1.35924	106-23-0	0.3±0.02a	0.19±0.08ab	0.14±0.06b	0.31±0.02a
Ethyl caprylate	1443.1	1283.926	1.48115	106-32-1	0.2±0a	0.13±0c	0.13±0c	0.18±0b
1-Octen-3-ol	1407.1	1200.008	1.15521	3391-86-4	0.53±0b	0.49±0.02b	0.59±0.01a	0.52±0.04b
hexyl butanoate	1396.7	1176.789	1.49036	2639-63-6	0.23±0b	0.23±0ab	0.24±0a	0.24±0ab
Heptyl acetate	1390.2	1162.583	2.02158	112-06-1	0.39±0.01a	0.35±0.02b	0.33±0.02b	0.32±0.01b
2-Octanol	1385.2	1151.608	1.80889	123-96-6	0.3±0	0.3±0	0.31±0	0.31±0
Allyl isothiocyanate	1342.1	1062.103	1.09882	57-06-7	0.26±0a	0.27±0a	0.07±0b	0.27±0a
Amyl isovalerate	1341.7	1061.346	1.4675	25415-62-7	0.35±0b	0.38±0.02a	0.03±0c	0.35±0.01b
2,6-Dimethylpyrazine	1306	992.452	1.5348	108-50-9	0.52±0b	0.49±0c	0.52±0b	0.54±0a
3-Hydroxy-2-butanone	1301.9	984.881	1.25585	513-86-0	0.16±0b	0.18±0a	0.09±0c	0.18±0a
Styrene	1301.6	984.367	1.43643	100-42-5	0.12±0b	0.17±0a	0.04±0c	0.17±0a
Hexyl acetate	1293.6	968.983	1.37874	142-92-7	0.15±0a	0.12±0b	0.11±0c	0.15±0a
1-Methyl-4-isopropylbenzene	1263.3	902.36	1.15832	99-87-6	0.25±0b	0.31±0a	0.16±0c	0.31±0a
Methylpyrazine	1251	876.62	1.38655	109-08-0	0.17±0b	0.21±0a	0.12±0c	0.21±0a
1,8-Cineol	1233.8	841.864	1.29517	470-82-6	0.31±0ab	0.31±0b	0.32±0a	0.32±0a
2-Pentylfuran	1202.9	782.886	1.24796	3777-69-3	0.18±0b	0.19±0ab	0.19±0a	0.19±0ab
2-Heptanal	1196.5	771.056	1.63387	110-43-0	0.23±0a	0.21±0b	0.21±0b	0.23±0a
α-Terpineol	1195.5	769.361	1.79135	10482-56-1	0.72±0.02a	0.68±0.02b	0.64±0.02bc	0.61±0.01c
1,8-Cineole	1192.1	763.251	1.32153	470-82-6	0.31±0ab	0.31±0b	0.32±0a	0.32±0a
3-Methyl-1-butanol	1185.1	746.313	1.51885	123-51-3	0.62±0b	0.61±0c	0.62±0b	0.63±0a
1,4-Cineol	1179.1	732.188	1.308	470-67-7	0.24±0b	0.24±0b	0.25±0ab	0.25±0a

1-Penten-3-ol	1150.2	667.416	1.36096	616-25-1	0.11±0.01a	0.13±0.01a	0.12±0.01a	0.11±0a
2-Methylpropyl butanoate	1145.2	656.795	1.8041	539-90-2	0.13±0.02a	0.08±0.01b	0.09±0.02b	0.08±0.02b
ethyl pentanoate	1130.2	625.996	1.2604	539-82-2	0.47±0ab	0.48±0.01a	0.48±0.01ab	0.46±0b
2,3-Pentanedione	1094	557.087	1.30586	600-14-6	0.23±0a	0.21±0ab	0.21±0ab	0.2±0.02b
3-Pentanol	1093.9	556.789	1.40793	584-02-1	0.19±0a	0.17±0b	0.19±0a	0.19±0a
2,3-dimethyl-5-ethylpyrazine	1092.9	554.852	1.74147	15707-34-3	0.98±0.02a	0.86±0.01b	0.11±0c	0.11±0c
2-Hexanone	1092.4	553.9	1.5343	591-78-6	0.1±0c	0.11±0b	0.12±0a	0.11±0b
Hexanal	1088.1	545.675	1.25024	66-25-1	0.57±0a	0.56±0.01a	0.57±0a	0.57±0a
1-Propanol, 2-methyl-D	1084.1	538.098	1.36427	78-83-1	0.97±0.01ab	0.99±0a	0.97±0ab	0.97±0b
1-Propanol, 2-methyl-M	1081.8	533.939	1.17374	78-83-1	0.13±0	0.14±0	0.15±0	0.15±0
2-methylbutanoic acid ethyl ester	1043.6	467.343	1.64832	7452-79-1	0.42±0a	0.32±0b	0.42±0.01a	0.43±0a
Methyl heptanoate	1029.1	444.306	1.3569	106-73-0	0.12±0bc	0.11±0c	0.13±0a	0.12±0ab
Ethyl 2-methylbutyrate	1028	442.622	1.64539	7452-79-1	0.42±0a	0.32±0b	0.42±0.01a	0.43±0a
Ethyl butanoate	1025.7	439.062	1.1991	105-54-4	0.44±0b	0.46±0ab	0.48±0.01a	0.46±0.01ab
Pyrrolidine	1021.7	432.929	1.26474	123-75-1	0.64±0a	0.63±0a	0.58±0b	0.63±0a
2-Pentanone-D	1019.3	429.322	1.37838	107-87-9	0.12±0.01b	0.07±0c	0.09±0bc	0.16±0.03a
2-Pentanone-M	1013.3	420.53	1.39839	107-87-9	0.1±0a	0.09±0b	0.09±0b	0.08±0c
1-Penten-3-one	1012.6	419.444	1.31352	1629-58-9	0.26±0b	0.27±0.01ab	0.29±0a	0.24±0.01c
2-butanol	987	384.883	1.33491	78-92-2	0.24±0d	0.26±0c	0.3±0a	0.28±0.01b
isobutyl acetate	986.5	384.276	1.60927	110-19-0	0.28±0d	0.35±0.01c	0.52±0a	0.45±0b
Pentanal	943.4	336.084	1.41725	110-62-3	0.14±0b	0.1±0c	0.09±0c	0.16±0a
2-Butanone	943.2	335.834	1.27218	78-93-3	0.27±0.01a	0.24±0b	0.23±0b	0.24±0b
Ethyl 2-methylpropanoate	936.6	328.961	1.31233	97-62-1	0.33±0a	0.3±0b	0.33±0a	0.33±0a
2,3-butanedione	934.4	326.688	1.5562	431-03-8	0.1±0c	0.66±0.01a	0.13±0b	0.12±0b
Ethyl propanoate	926.8	319.11	1.44894	105-37-3	0.88±0.01a	0.74±0.01c	0.77±0.01b	0.88±0.01a

1,1-diethoxy ethane	894.1	288.195	1.20218	105-57-7	0.43±0.01b	0.38±0c	0.46±0.01a	0.42±0b
3-Methylbutanal	877.6	273.71	1.40211	590-86-3	0.1±0ab	0.07±0bc	0.04±0c	0.13±0.03a
1,3-Butanediol	876.2	272.504	1.3681	107-88-0	0.31±0a	0.26±0b	0.17±0c	0.35±0.04a
Ethyl acetate	860.2	259.249	1.33153	141-78-6	0.26±0	0.27±0	0.27±0	0.27±0
butanal	837.5	241.555	1.09347	123-72-8	0.35±0b	0.37±0a	0.36±0b	0.36±0b
Ethyl formate	832.4	237.744	1.22173	110-62-3	0.14±0b	0.1±0c	0.09±0c	0.16±0a
propyl propanoate	811.6	222.808	1.57685	106-36-5	0.09±0.01a	0.09±0.01a	0.09±0a	0.07±0.01b
Triethylamine	811.6	222.861	1.46722	121-44-8	0.09±0.01a	0.09±0.02a	0.09±0a	0.07±0.01a
propanal	798.9	214.178	1.02855	123-38-6	0.69±0b	0.69±0.01b	0.8±0.01a	0.68±0b
Acetic acid, methyl ester	796.2	212.389	1.18801	79-20-9	0.79±0.01a	0.76±0.01b	0.11±0d	0.74±0c
2,4-dimethylheptane	794.2	211.069	1.20637	2213-23-2	0.47±0b	0.39±0d	0.43±0.01c	0.5±0.01a
2-methyltetrahydrofuran-3-one	793.6	210.656	1.06707	3188-00-9	0.3±0a	0.3±0a	0.26±0b	0.31±0.01a
Acetone	782.6	203.579	1.11372	67-64-1	0.16±0ab	0.16±0ab	0.15±0.01b	0.17±0.01a
Isopentyl formate	774.6	198.542	1.27569	110-45-2	0.22±0b	0.08±0b	0.11±0b	0.39±0.12a
Cyclohexane	760.7	190.127	1.04208	110-82-7	0.37±0.01b	0.52±0.01a	0.49±0a	0.34±0.04b
1-heptene	742.8	179.806	1.08554	592-76-7	0.08±0b	0.09±0ab	0.08±0b	0.1±0.01a
1,1-diethoxyethane	719.6	167.296	1.13064	105-57-7	0.43±0.01b	0.38±0c	0.46±0.01a	0.42±0b
Propionic acid	716.8	165.86	1.11705	79-09-4	0.23±0.01a	0.17±0.02b	0.18±0.01b	0.16±0.04b
(E)-3-Pentenenitrile	693.8	154.376	1.15433	16529-66-1	0.13±0c	0.14±0bc	0.14±0ab	0.15±0.01a
Area 1	2275	2312	1.3858		0.35±0c	0.4±0a	0.38±0.01b	0.31±0d
Area 2	1137	1192	1.2609		0.62±0a	0.59±0	0.61±0.01ab	0.62±0.02ab
Area 3	1218	1247	1.2288		0.23±0b	0.19±0c	0.18±0.01c	0.27±0.01a
Area 4	757	783	1.2213		0.3±0.01b	0.19±0c	0.18±0.01c	0.38±0.01a
Area 5	721	731	1.2158		0.21±0b	0.17±0c	0.18±0.01c	0.25±0.01a
Area 6	688	726	1.1595		0.13±0b	0.09±0c	0.08±0c	0.17±0.02a
Area 7	744	771	1.167		0.3±0.01a	0.33±0.04a	0.37±0.05a	0.35±0.05a

Note: RI represented relative retention index; Rt represented retention time; Dt represented relative migration time; The suffixes -M represented monomer while the suffixes -D represented dimer; values with different letters (a-d) in a row were significantly different using Duncan's multiple comparison tests ($p < 0.05$).

Table S2. Presents the odor activity values (OAV ≥ 1) for GAB fermented by different *Saccharomyces cerevisiae* strains

Compound	Odor threshold ($\mu g \cdot L^{-1}$)[a]	Aroma description[b]	OAV[c]			
			SY	EC-1118	NM-8	ET008-c54
3-Methyl-1-Butanol	600	alcohol, banana, cheese, cocoa, floral	1.85	1.58	1.48	2.00
Phenylethyl Alcohol	564.23	corn flakes, floral, fruit, honey, rose	1.20	1.09	<1	1.27
Octanoic Acid	500	acid, carrot, cheese, fat, fermented	1.80	1.73	1.41	1.32
β-Lonone	0.007	cedarwood, dry, floral, raspberry, seaweed	1107.14	825.71	944.29	982.86
Irisone	0.45	floral, sweet, violet	ND	14.96	ND	5.96
Ionene	2	floral	3.08	2.94	2.65	3.57
Ethyl Caprate	200	brandy, burnt, fruit, geranium, grape	4.21	3.72	2.91	4.29
Ethyl Caprylate	5	apricot, banana, brandy, fresh, fruit	105.85	82.45	69.67	85.23
Ethyl laurate	400	cream, floral, fruit, green apple, leaf	1.06	<1	<1	<1
Ethyl hexanoate	14	anise, apple peel, banana, brandy, cheese	9.32	8.09	6.68	5.46
ethyl butanoate	0.9	anise, apple, banana, butter, fruit	8.43	6.21	6.32	5.31
Ethyl isobutyrate	0.1	apple, floral, fruit, rubber, strawberry	16.90	6.70	24.60	20.80
Ethyl 2-methylbutanoate	0.2	anise, apple, bubble gum, floral, fruit	8.70	6.05	8.60	8.45

[a] Odor threshold value in water from Thresholds are taken from the literature and a database: www.vcf-online.nl; “NF”: not found..

[b] Odor descriptions were reported at http://www.vcf-online.nl.

[c] OAV was calculated based on average concentrations of volatile compounds in Table. 2.
